# Motor Vehicle Collision Involvement among Persons with Hemianopia and Quadrantanopia

**DOI:** 10.3390/geriatrics1030019

**Published:** 2016-07-18

**Authors:** Gerald McGwin, Joanne Wood, Carrie Huisingh, Cynthia Owsley

**Affiliations:** 1 Department of Ophthalmology, School of Medicine, University of Alabama at Birmingham, 1720 University Boulevard, Suite 609, Birmingham, AL 35294, USA; huisingh@uab.edu (C.H.); owsley@uab.edu (C.O.); 2 Department of Epidemiology, School of Public Health, University of Alabama at Birmingham, Birmingham, AL 35294, USA; 3 School of Optometry and Vision Science, Queensland University of Technology, Brisbane, Queensland 4059, Australia; j.wood@qut.edu.au; 4 Institute of Health and Biomedical Innovation, Queensland University of Technology, Brisbane, Queensland 4001, Australia

**Keywords:** automobile driving, visual fields, hemianopsia

## Abstract

Persons with homonymous quadrantanopia and hemianopia experience driving restrictions, yet there is little scientific evidence to support driving prohibition among persons with these conditions. This retrospective cohort study compares motor vehicle collision (MVC) rates among 27 current licensed drivers with hemianopic and quadrantanopic field defects, who were ≥6 months from the brain injury date with that of 27 age-matched drivers with normal visual fields. Information regarding all police-reported MVCs that occurred over a period of nine years was obtained. MVC rates per year and per mile travelled were calculated and compared using conditional Poisson regression. Drivers with hemianopia or quadrantanopia had more MVCs per mile driven compared to drivers with normal visual fields; specifically their overall MVC rate was 2.45-times (95% confidence interval (CI) 0.89–3.95) higher and their at-fault MVC rate was 2.64-times (95% CI 1.03–6.80) higher. This study indicates that drivers with hemianopia or quadrantanopia have elevated MVC rates. This is consistent with previous research despite studies showing wide individual variability from excellent to poor driving skills. Future research should focus on the functional and driving performance characteristics associated with superior driving skills and/or those that may be amenable to improvement via behavioral and/or engineering interventions.

## 1. Introduction

Persons with homonymous quadrantanopia and hemianopia experience visual field defects where one-quarter or one-half of the binocular visual field, respectively, is absent. These defects result from post-chiasmal injury to the visual pathways and are typically secondary to acute events such as a stroke or traumatic brain injury or the result of a tumor [1]. The most common cause of hemianopia in older adults is stroke [2]. As the population ages, the incidence of stroke and subsequent hemianopia is likely to increase [3]. Persons with these conditions commonly report mobility limitations and reduced quality of life, including driving problems [4,5]. A better understanding how adults manage these visual difficulties, especially while driving, is needed.

Driving limitations for those with hemianopic or quadrantanopic field loss reflect both self- and government-imposed restrictions, including complete denial of driving privileges in many jurisdictions around the world [6]. Yet as with many vision standards for licensure, there is little scientific evidence to support driving prohibition among persons with these conditions [7]. While decrements in driving skills have been observed for some drivers with hemianopia or quadrantanopia in both on-road studies [8,9,10,11,12] and driving simulators [13,14,15,16,17,18,19], recent studies suggest that some persons with quadrantanopia and hemianopia actually display driving skills that are indistinguishable from drivers with normal visual fields [9,10,11,15]. It is also important to consider evidence suggesting that drivers with hemianopia and quadrantanopia adopt visual behaviors that compensate for their vision impairment when engaging in tasks in dynamic and unpredictable environments including driving [11,20,21], thereby possibly minimizing the likelihood of crash involvement. However, to our knowledge, there are no published reports of the motor vehicle collision (MVC) risk of individuals with hemianopic or quadrantanopic field defects. Therefore, the objective of the current study is to compare motor vehicle collision involvement rates between drivers with hemianopia or quadrantanopia and age-matched individuals with normal visual fields.

## 2. Materials and Methods

The study design and procedures have been previously described [9,10,11] and are briefly summarized below.

### 2.1. Participants

Potential homonymous hemianopic and quadrantanopic participants were identified through the Neuro-Ophthalmology service of the Department of Ophthalmology clinic at the University of Alabama at Birmingham (UAB) through ICD-9 codes 368.46 (homonymous bilateral field defects), 368.47 (heteronymous bilateral field defects) and 368.40 (visual field defect, unspecified) for two years retrospective to the study start date. Of the 802 potential participants with hemianopia or quadrantanopia identified, 70 were excluded because their medical records were unavailable (e.g., archived to a remote site). Of the remaining 732 medical records reviewed, 55 met the eligibility criteria after chart review. Common reasons for ineligibility based on medical record review were the person did not have homonymous hemianopia or quadrantanopia, had given up driving permanently or had not returned to driving post-brain injury, paralysis, or had medical conditions that were exclusion criteria (e.g., Alzheimer’s disease, glaucoma). Of the 58 eligible patients, 27 persons with hemianopia or quadrantanopia enrolled in the study. Reasons for not enrolling included deceased (*n* = 4), could not be contacted (*n* = 4), and declined participation (*n* = 20). They were contacted by a letter from their neuro-ophthalmolgist describing the study, and those interested were scheduled for participation. Persons with normal visual fields were recruited from a list of volunteers interested in research participation in the Clinical Research Unit at UAB. In order to create an age-matched reference group, as each hemianopic or quadrantanopic participant was enrolled, an individual from the research volunteer database was selected whose age was ±2 years of the age of the hemianopic or quadrantanopic participant just enrolled.

Inclusion criteria for all participants were aged ≥19 years old, visual acuity of 20/60 or better in at least one eye, were legally licensed to drive in the State of Alabama and were a current driver. Exclusion criteria were diagnoses of Parkinson’s disease, multiple sclerosis, Alzheimer’s disease, hemiparesis and other types of paralysis, or an ophthalmic or neurologic condition characterized by visual field impairment (other than hemianopia or quadrantanopia for the visual field loss group), the requirement for adaptive equipment in a vehicle in order to drive, and lateral spatial neglect as determined by the Stars test [22]. Additional inclusion criteria for hemianopic and quadrantanopic participants were homonymous hemianopic or quadrantanopic visual field defect as indicated by the most recent visual field assessment in the medical record and ≥6 months from the brain injury date. Those persons in the age-matched reference group were required to have normal visual fields (see below), and they could have no history of brain injury (e.g., stroke, trauma, tumor, arteriovenous malformation).

This study was conducted in accordance with the Declaration of Helsinki and the protocol was approved by the Institutional Review Board for Human Use at the University of Alabama at Birmingham. After the purpose of the study was explained, participants were asked to sign a document of informed consent before enrolling.

### 2.2. Procedures

Demographic information (age, gender, race) was obtained by medical record review and confirmed by interview. The number of co-morbid medical conditions was estimated using a general health questionnaire which has been used extensively in previous studies [23]. Participants were asked to report all prescription and non-prescription medications they were taking. The Driving Habits Questionnaire [24] was used to confirm driving status and licensure and estimate driving exposure (days/week, miles/week driven) in the recent past. General cognitive status was screened using the Mini-Mental Status Examination (MMSE) [25]. All questionnaires were interviewer-administered by trained staff.

Visual acuity was assessed binocularly using the standard protocol of the Early Treatment for Diabetic Retinopathy Study chart [26] and expressed as logMAR. Binocular letter contrast sensitivity was measured using the Pelli-Robson chart under the recommended testing conditions [27] and scored by the letter-by-letter method [28]. Trail Making Tests A and B were used to examine visual search, processing speed, mental flexibility, and executive function [29]. Processing speed, short-term memory, and attention switching were measured using the Digit Symbol Substitution test (DSST) [30]. Visual fields were assessed monocularly and binocularly using automated static perimetry (Humphrey Field Analyzer Model 750i, Carl Zeiss Meditec, California, US). Right and left monocular fields were measured using the central threshold 24-2 test with the SITA test strategy. Binocular visual fields were measured using the Binocular Esterman test with participants wearing the refractive error correction usually worn when driving, if any. Results were used to confirm the presence of homonymous hemianopia, quadrantanopia, or normal visual fields.

Information regarding all MVCs that occurred between January 2002 and October 2010 wherein the study participant was the driver was obtained from the Alabama Department of Public Safety. Information of specific relevance to the current study was abstracted from hard-copy accident reports, including the date of the MVC and whether the study participant was deemed at-fault according to the officer at the scene. For hemianopic and quadrantanopic participants diagnosed after 2002, only those MVCs that occurred post-diagnosis were included in the analysis.

### 2.3. Statistical Analysis

T- and Fisher’s exact tests were used to compare the field loss groups with respect to continuous and categorical variables, respectively. The combined hemianopic and quadrantanopic group was compared to the normal visual field group using paired t- and McNemar’s test to account for the pair matched nature of the study design. MVC rates were calculated per year and per mile driven. With respect to the former, follow-up time was calculated as the duration of time between January 2002 or, for hemianopic and quadrantanopic participants their date of diagnosis if it was after January 2002, and October 2010 or the date of driving cessation. Thus, for each study participant, the maximum follow-up time was approximately 8.8 years. For the calculation of mileage-based MVC rates, each participant’s follow-up time was multiplied by their self-reported annual mileage in order to obtain an estimate of the total distance driven during the period of observation, under the assumption that the mileage reported at baseline has remained constant over the period of observation. Conditional Poisson regression was used to compare MVC rate per year and per mile driven between the hemianopic and quadrantanopic participants compared to participants with normal visual fields. *p*-values of ≤0.05 (two-sided) were considered statistically significant.

## 3. Results

The sample consisted of 20 participants with hemianopic field loss, seven with quadrantanopic field loss and 27 age-matched participants with normal visual fields; the average (median) follow-up time was 6.7 (7.5) years, 6.5 (6.0) years and 8.8 (8.8) years, respectively. The etiology of hemianopic defects was 11 participants with cerebrovascular accident (CVA), three head trauma, two arteriovenous malformation, two tumor, one congenital brain anomaly, and one lobectomy as treatment for epilepsy. The etiology of quadrantanopic defects was five participants with CVA, one tumor, and one congenital anomaly. Table 1 presents the demographic, health (including visual and cognitive), and driving characteristics for the study participants. One-third (*n* = 18) were aged ≥60 years. There were no significant differences in these characteristics between the hemianopic and quadrantanopic groups. However, when the two field loss groups were combined, the participants with field loss were significantly more likely to be male and to be in poorer health as indicated by the number of chronic medical conditions and current medications. Despite significant differences in visual function in the two groups, visual acuity averaged 20/25 or better and contrast sensitivity was high (averaging 1.7–1.8) in both groups. Field loss participants also had significantly worse MMSE scores though all participants scored in the non-demented range (i.e., ≥24) and the average MMSE scores in each group were only separated by one point. Visual processing speed and attentional skills as assessed by Trails A, Trails B and the DSST were significantly worse in those with hemianopia or quadrantanopia compared to the group with normal fields. Finally, field loss participants reported lower weekly mileage compared to those with normal visual fields. 

Table 2 presents the MVC outcomes for the three groups of study participants. With respect to the gender-adjusted rate ratios (RRs) based on person-time, overall and at-fault MVC rates were 1.1- and 2.5-times higher, respectively, among hemianopia and quadrantanopia participants compared to those with normal visual fields; however, neither of these associations was statistically significant, possibly owing to the small sample size. When considered individually, both hemianopic and quadrantanopic participants had elevated, though non-significant, at-fault RRs. When based on person-mileage, hemianopia and quadrantanopia participants combined had a 2.5- and 2.6-fold increased overall and at-fault MVC rates, respectively; the latter association being statistically significant. Each group alone also had elevated RRs though these were significant only for the hemianopia participants.

With the exception of demographic characteristics (i.e., gender), the other measurements in Table 1, to a certain degree, are consequences of hemianopia and quadrantanopia rather than potential confounders. Though, in some settings, these same measurements would be subject to statistical adjustment, this is not necessary in the current context; in fact doing so would introduce bias into the measures of association.

## 4. Discussion

The results of the current study suggest that drivers with visual field loss attributable to homonymous hemianopia and quadrantanopia have elevated MVC rates compared to similarly aged drivers with normal visual fields, when rates are defined in terms of person-miles of driving. This is the first study of state-recorded MVC in drivers with hemianopic and quadrantanopic field loss; however, there are a number of studies that have sought to evaluate driving performance, both in a simulator and on-road. Using a driving simulator, Szlyk et al. found that the driving performance of older adults with homonymous hemianopia or quadrantanopia secondary to stroke was worse than that of drivers with normal vision, particularly with respect to lane boundary crossings [13]. However, a similar study reported no differences in driving speed, reaction time, or driving errors between a group of patients with cerebral field defects (mostly homonymous binocular defects) and a normally sighted control group [31]. In a series of reports [9,10,11], the real-world, on-road driving performance of 22 patients with hemianopic field loss and eight patients with quadrantanopic field loss was compared to a normally sighted age-matched control group. Elgin et al. reported that the visual field loss patients had more driving errors than the control group; specifically there were marked differences with vehicle and speed control, reaction to unexpected events, and bad driving maneuvers (e.g., stopping in a lane on the interstate) [10]. In a separate study based upon the same patients, the visual field loss patients reported more difficulty with driving maneuvers requiring peripheral vision (e.g., left-hand turns across traffic) and with independent mobility (e.g., driving in unfamiliar areas) [5]. In another study, the field loss patients performed significantly worse with respect to lane position and steering steadiness; however, the masked, back-seat raters also determined that the majority of patients had the potential for safe driving [9]. Using a driving simulator, Bowers et al. compared the detection of pedestrians [14], lane position, and steering [32] between a group of 12 homonymous hemianopia patients and an age-matched group with normal vision. The hemianopia patients had lower pedestrian detection rates. Papageorgiou et al. reported more collisions in a virtual-reality driving simulator among the field loss group compared to normal controls [15]. 

It would be inappropriate to conclude from the results of this study that persons with hemianopia should automatically be denied driver’s licenses. First, the MVC rates for drivers with hemianopic and quadrantanopic field loss reported in this study are not dissimilar from those reported for drivers with other medical conditions or disorders (e.g., cataract), for whom driving is not explicitly prohibited in most jurisdictions in the U.S. [33] In fact, the National Highway Traffic Safety Administration has concluded that licensing agencies should impose restrictions based upon functional impairments in an attempt to maintain a balance between the transportation needs of drivers with medical conditions and public safety. Second, this is the first study to address MVCs among drivers with hemianopia and therefore the results require replication. Third, as noted above, like all studies in this area, it is based upon a small number of patients and as a consequence the measures of association lack precision. Thus, though the elevated MVC rates are statistically significant and some are of moderate strength, the magnitude of the true association lies within a wide range from weak to strong. Fourth, a less methodological and more practical consideration relates to attributable risk; that is, despite the elevated MVC rate, the prevalence of hemianopia is sufficiently low that the number of MVCs prevented by removing these patients from the road will be small and not likely outweighed by the mobility and psychological (e.g., depression) consequences associated with driving cessation [34]. Denying driver’s licenses to hemianopia patients outright ignores the fact, that despite having worse driving skills on average, some are competent drivers. This fact is reflected in recommendations that driving fitness among persons with hemianopia be evaluated on an individual basis using an on-road evaluation as part of that assessment [8,9,10,35]. Moreover, there is emerging evidence that can be used to guide such assessments. Wood et al. have identified several clinical and driving performance characteristics associated with a rating of unsafe driving (based upon an on-road driving assessment) among hemianopia and quadrantanopia patients [9,11]. Others have reported related findings from simulator studies that may also be used to inform such driving assessments [14,15]. Importantly, several recent studies have reported that some drivers with hemianopic loss demonstrate similar driving abilities to those of normal subjects through the development of compensatory eye and head movements [11,15,36]. It has also been suggested that such research could be used to inform the development of interventions (e.g., scanning training, prism glasses) directed towards improving driving-related skills deficient in this patient population [11,14].

## 5. Conclusions

Consistent with prior research, the results of this study indicate that drivers with hemianopia and quadrantanopia are less proficient drivers than similarly aged drivers without visual field impairments. Future research involving larger numbers of patients should seek to determine those functional and driving performance characteristics that could be used to identify high-risk patients whose driving skills may be amenable to improvement via behavioral and/or engineering interventions.

## Figures and Tables

**Table 1:** Demographic, medical, functional and driving characteristics among drivers hemianopic or quadrantanopic field loss and age-matched drivers with normal visual fields.

	Participants with Field Loss *N* = 27			Participants with Normal Fields *N* = 27
	Hemianopia *N* = 20	Quadrantanopia *N* = 7	Combined *N* = 27	
**Age, years, mean (sd)**	50.5 (19.6)	50.7 (18.5)	50.5 (19.0)	50.4 (18.3)
**Gender, %**				
**Male**	60.0	71.4 *	63.0*	29.6
**Race, %**				
**African American**	10.0	0.0	7.4	14.8
**White, non-Hispanic**	85.0	100.0	89.9	85.2
**Other**	5.0	0.0	3.7	0.0
**# Chronic medical conditions, mean (sd)**	5.6 * (3.3)	4.3 (1.3)	5.2 * (2.9)	2.1 (1.5)
**# Current medications, mean (sd)**	5.2 * (4.1)	4.6 (3.7)	5.0 * (3.9)	2.2 (2.1)
**Visual acuity, OU, logMAR, mean (sd)**	0.07 * (0.30)	−0.02 (0.35)	0.04 * (0.31)	−0.17 (0.22)
**Contrast sensitivity, OU, log sensitivity, mean (sd)**	1.75 (0.17)	1.81 (0.13)	1.76 * (0.16)	1.85 (0.10)
**MMSE score, mean (sd)**	28.4 (1.6)	28.7 (0.8)	28.4 * (1.5)	29.2 (1.2)
**Estimated weekly mileage, mean (sd)**	177.4 (133.7)	182.1 (111.3)	178.6 * (126.2)	296.7 (215.3)
**Time since injury, years, mean (sd)**	8.0 (13.4)	11.4 (20.5)	9.0 (15.2)	NA

* *p* ≤ 0.05 compared to participants with normal fields. sd, standard deviation; #, number of; NA, Not applicable.

**Table 2:** Gender adjusted rate ratios (RRs) and 95% confidence intervals (CIs) comparing motor vehicle collision (MVC) rates between drivers hemianopic or quadrantanopic field loss and age-matched drivers with normal visual fields.

	Participants with Field Loss *N* = 27			Participants with Normal Fields *N* = 27
	Hemianopia *N* = 20	Quadrantanopia *N* = 7	Combined *N* = 27	
**Motor vehicle collisions**				
**All**	15	4	19	21
**At-fault**	10	2	12	10
**Person-years**	134.6	45.8	180.4	238.4
**All MVC RR (95% CI)**	1.61 (0.65-3.96)	0.37 (0.08–1.70)	1.10 (0.51–2.36)	---
**At-fault MVC RR (95% CI)**	3.13 (0.78–12.46)	0.83 (0.04–17.72)	2.50 (0.76–8.21)	---
**Person-miles**	1,098,917	419,296	1,518,213	3,678,181
**All MVC RR (95% CI)**	2.43 (1.05–5.62)	0.87 (0.20–3.73)	2.45 (0.89–3.95)	---
**At-fault MVC RR (95% CI)**	2.69 (1.00–7.30)	2.31 (0.17–31.22)	2.64 (1.03–6.80)	---

## Abbreviations

CI	Confidence interval
CVA	Cerebrovascular accident
DSST	Digit symbol substitution test
MMSE	Mini-mental status examination
MVC	Motor vehicle collision
N	Number
NA	Not applicable
OU	Oculus uterque for both eyes
RR	Rate ratio
SD	Standard deviation
UAB	University of Alabama at Birmingham
